# Chromatin Dynamics in Genome Stability: Roles in Suppressing Endogenous DNA Damage and Facilitating DNA Repair

**DOI:** 10.3390/ijms18071486

**Published:** 2017-07-10

**Authors:** Nidhi Nair, Muhammad Shoaib, Claus Storgaard Sørensen

**Affiliations:** Biotech Research and Innovation Centre (BRIC), University of Copenhagen, Ole Maaløes Vej 5, Copenhagen N 2200, Denmark; nidhi.nair@bric.ku.dk (N.N.); muhammad.shoaib@bric.ku.dk (M.S.)

**Keywords:** genome maintenance, chromatin, DNA damage response, endogenous DNA damage

## Abstract

Genomic DNA is compacted into chromatin through packaging with histone and non-histone proteins. Importantly, DNA accessibility is dynamically regulated to ensure genome stability. This is exemplified in the response to DNA damage where chromatin relaxation near genomic lesions serves to promote access of relevant enzymes to specific DNA regions for signaling and repair. Furthermore, recent data highlight genome maintenance roles of chromatin through the regulation of endogenous DNA-templated processes including transcription and replication. Here, we review research that shows the importance of chromatin structure regulation in maintaining genome integrity by multiple mechanisms including facilitating DNA repair and directly suppressing endogenous DNA damage.

## 1. Introduction

Cellular genome holds the blueprint of life; hence, a fundamental evolutionary goal of organisms is to preserve the integrity of their genomes. Throughout the lifespan of an organism, their genome is constantly under threat from exogenous agents, endogenous insults and cellular processes that have the capability to inflict damage and severely compromise genome integrity. In this regard, cells have developed evolutionarily conserved defense mechanisms to counteract the detrimental effects of these events and safeguard the genetic information. An important mechanism is the DNA damage response (DDR), which is a complex signal transduction pathway that has the ability to sense DNA damage and transduce this information to influence cellular responses to DNA damage and promote repair [1,2]. In higher eukaryotes, the DDR has been extensively studied in the context of DNA double strand breaks (DSBs), where it comprises of two key responses: (i) the rapid activation of cell cycle checkpoints; and (ii) recruitment of DNA-repair proteins onto the chromatin at the DSB site. In this regard, the first responder is the MRN (MRE11, RAD50, and NBS1) complex, which functions to recruit and activate the ATM (Ataxia Telangiectasia mutated) protein kinase [2]. The DDR occurs in the context of chromatin and its efficiency is heavily dependent upon chromatin structure. ATM kinase phosphorylates several downstream targets with a major target being histone H2A variant H2A.X referred to as γH2A.X [3]. Phosphorylated H2A.X reaches up to two megabases around the DSBs and initiates a cascade of repair factor recruitment [1,2].

**Figure 1:**
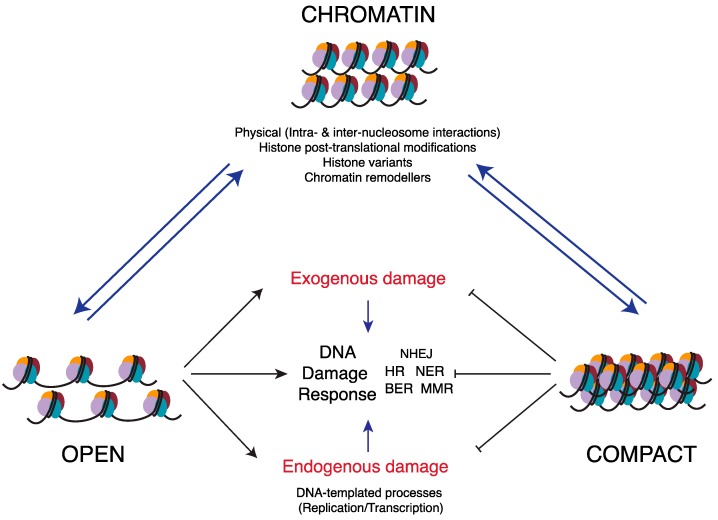
Chromatin structure dynamics regulate genome stability. Chromatin structural organization is dynamically controlled by intra- and inter-nucleosomal interactions, histone post-translational modifications (PTMs) and histone variants, and activity of ATP-dependent chromatin remodelers. All these factors ensure proper chromatin conformation during various stages of cell cycle and during various DNA-templated process. A more relaxed or “OPEN” chromatin conformation is prone to both exogenous and endogenous damage while at the same time leads to enhanced DNA damage response (DDR). The more compact or “CLOSED” chromatin conformation suppresses both kinds of genomic insults, however, it is generally inhibitory to DDR. Apart from DNA repair, the “CLOSED” chromatin conformation is also inhibitory to other DNA-templated processes such as transcription and replication. Therefore, the dynamic nature of chromatin structure provides a way to not only repair DNA lesions but also allows access to cellular machineries to perform DNA-based processes and in turn maintain genome stability. NHEJ; non-homologous end joining, HR; homologous recombination, NER; nucleotide excision repair, BER; base excision repair, MMR; mismatch repair.

Importantly, the complexity and diversity in chromatin organization influences genome stability at several levels. This includes the suppression of endogenous damage by promoting fine-tuned DNA templated processes such as DNA replication and transcription in a spatio-temporal manner during various cell cycle phases. However, chromatin structure also poses a significant obstacle to detection and repair of DNA lesions. In this regard, plasticity of chromatin structure plays a central role in facilitating and regulating cellular response to DNA damage [4,5]. Moreover, chromatin structure actively shields the DNA template and poses a physical barrier to exogenous insults on the DNA structure and sequence [6,7,8] (Figure 1). In this review, we summarize research highlighting the interplay between chromatin structural organization and genome stability. 

## 2. Chromatin Structural Landscape

The key determinant of nuclear organization is evolutionarily conserved packaging of genome into chromatin, a hierarchically organized complex of DNA, histones and non-histone proteins [9]. At the simplest level, DNA is wrapped around nucleosomes consisting of an octamer of core histones, namely two molecules each of histones H2A, H2B, H3 and H4. Nucleosomes represent the fundamental repeating unit of eukaryotic chromatin and are connected by a short linker DNA segment with the help of linker histone H1 and grouped in discrete domains, which interact with neighboring nucleosomes and form chromatin fibers. The most basic linear organization of individual nucleosomes (DNA wrapped around histones) in a so-called “beads on a string” can be regarded as the “primary” structure of chromatin fiber [10]. The number of nucleosomes in each chromatin domain determines the density of chromatin fiber and gives rise to “secondary” and “tertiary” higher order chromatin structures [9,10,11]. The secondary structures arise as a result of inter-nucleosomal interactions while tertiary structures represent further interactions between secondary structures to form large chromatin domains [10,12]. Furthermore, the stability of secondary and tertiary structures is dependent upon architectural proteins, such as linker histone H1, methyl-CpG-binding protein 2 (MeCP2), heterochromatin protein 1 (HP1), high mobility group (HMG) proteins, poly(ADP-ribose) polymerase 1 (PARP1), myeloid and erythroid nuclear termination stage-specific protein (MENT), Polycomb group proteins and many others. On the basis of differential compaction and functional status, interphase nuclei in eukaryotic cells have been shown to contain two distinct forms of chromatin that is euchromatin, a rather relaxed and transcriptionally active conformation, and a more compacted and transcriptionally inactive form named heterochromatin [13]. Heterochromatin is further classified as constitutive and facultative heterochromatin. Constitutive heterochromatin generally enriched in repetitive, gene-poor, and late replicating DNA sequences and is invariably compact, whereas facultative heterochromatin is more dynamic in nature as it can reversibly undergo transitions from a compact, transcriptionally silent state into a more open, and transcriptionally active state [10,14]. The distinction in chromatin structure can also be described by complex histone post-translational modifications (PTMs) patterns (Table 1) that specify distinct chromatin states via several different combinations of modifications as well as the proteins that associate with them. For example, histone H3 di/tri-methylated on lysine 9 (H3K9me2/3) and histone H4 lysine 20 tri-methylation (H4K20me3) marks are abundant in pericentric constitutive heterochromatin while acetylation of histone H3 lysine 9 and H4 N-terminal lysine residues is characteristic of a more relaxed chromatin state [15].

## 3. Genomic Instability in Heterochromatic Repetitive DNA Sequences

A defining genomic characteristic of heterochromatin is that it is highly enriched in repetitive sequences and concentrated in pericentromeric and telomeric regions in most eukaryotes [16]. These repetitive sequences include highly repeated tandem “satellite” sequences (centromeric, and micro- and mini-satellites) and transposable elements (DNA and RNA transposons), generally have relatively low gene density, and vary in DNA size from five to few hundred basepairs [17]. The structural and functional regulation of DNA repeats and transposable elements is crucial for maintaining genome integrity. Repetitive DNA sequences are highly prone to DNA DSBs and may be hotspots for meiotic crossover and other recombination events, some of which can cause genome instability [18]. Recombination between repetitive DNA sequences frequently results in chromosome rearrangements, a hallmark of cancer and hereditary disorders [17,19]. In this regard, heterochromatin formation at repetitive elements is a protective mechanism evolved for suppressing aberrant recombination events by prohibiting illegitimate recombination between dispersed repetitive DNA elements [17]. Thus, disruption of SU(VAR)3-9H1/2 (Suppressor of variegation 3–9 homolog 1/2)-dependent H3K9 methylation at pericentric heterochromatin severely impairs viability and induces chromosomal instabilities [20,21]. Importantly, in drosophila it was observed that highly repetitive rDNA is associated with heterochromatin where loss of SU(VAR)3-9 and accompanying histone PTMs (H3K9me2/3) leads to nucleolar disruption and a concomitant increase in extrachromosomal circular DNA due to aberrant recombination events [22]. This suggests that heterochromatin associated with rDNA repeats may serve to suppress accumulation of challenging DNA structures while potentially also limiting recombination aberrations to maintain genome stability.

Furthermore, transposable elements owing to their intrinsic property of being excised and subsequently inserted elsewhere in the genome have the potential to remodel gene structure and expression by being directly inserted into either the coding sequence or the regulatory elements of genes [17,23]. Compaction of chromatin over transposable elements therefore, prevents their activation and spreading and in turn prevents genome instability. The heterochromatic silencing has been suggested as an evolutionary measure to control and limit the unrestricted movement of transposable elements across the genome. In general, the repetitive elements are silenced by three epigenetic pathways, i.e., methylation of H3K9, DNA methylation, and the PIWI pathway, the latter active mainly during developmental stages [17]. An inverse correlation has been found between the levels of histone and DNA methylation as well as capability of high transcriptional and transpositional activity of different repeat families [24,25]. It is however unclear at the moment if different repeat families have the same capacity to inflict genome instability in an event of loss of their heterochromatinization.

Aberrant overexpression of pericentromeric satellite repeats have been observed in lung cancer while decompaction and transcriptional activation of pericentromeric heterochromatin has been reported in several genetic disorders [26,27]. Similarly, downregulation of Lysine-specific demethylase 2A (KDM2A) in prostate cancer leads to aberrations in compaction status and activation of pericentromeric repeats. KDM2A is a H3K36 specific demethylase and is actively targeted to pericentromeric heterochromatin and functions to compact chromatin structure at those repeats. Loss of KDM2A results in decompaction and transcriptional activation of these heterochromatic elements leading to chromosome segregation defects and genome instability [28]. BRCA1 (Breast cancer susceptibility protein 1) tumor suppressor has also been linked to repression of tandem repeat satellite DNA probably through monoubiquitylation of histone H2A at lysine 119 (H2AK119Ub). It has been shown that loss of BRCA1 leads to decrease in the condensed regions of genome and loss of H2AK119Ub at satellite repeats [29]. The derepression of pericentromeric satellite DNA leads to global loss of heterochromatin integrity and invariably leads to mitotic defects and increased DNA double-strand breaks [29].

In order to ensure genome stability and to prevent illicit recombination between the vast amounts of repetitive DNA, heterochromatic structures must be faithfully replicated. In this regard, several chromatin-remodeling enzymes, such as SMARCA5 (SWI/SNF-related matrix-associated actin-dependent regulator of chromatin subfamily A member 5) ensure efficient replication of heterochromatic late replicating domains most likely by facilitating chromatin remodeling to allow the progression of the replication fork [30]. Furthermore, SMARCAD1 protein, a protein related to the SWI/SNF family of nucleosome remodelers, has also been shown to promote histone deacetylation after their deposition by replicative chaperones in order to maintain a repressive and compact chromatin structure right after heterochromatin replication [31]. Fun30, the yeast homolog of human SMARCAD1, has been shown to promote silencing by regulating the chromatin structure within transcriptionally repressed domains [32], however, it is not known whether loss of SMARCAD1 leads to increased transcription of repressed genome and subsequent genome instability. Additionally, nucleosome remodeling and deacetylase complex (NuRD) has been shown to promote genome stability presumably by its involvement in preserving higher order chromatin structures. In this regard, the loss of NuRD complex components RBBP4, RBBP7 and HDAC1 compromises the establishment and maintenance of histone modifications and higher-order chromatin architecture. It was suggested that such loss of heterochromatin structure contributed to genome instability by making chromatin more susceptible to DNA damage [33]. Alternatively, impaired DNA replication might also contribute to the loss of genome stability in heterochromatic, late replicating regions of the genome [16].

## 4. Chromatin Structure and Repair of Endogenous Lesions

Human cells are subjected to a daily load of approximately 70,000 genomic lesions [34], most of which are of endogenous origin. Major sources of endogenous DNA damage include reactive oxygen species (ROS)-dependent lesions, aldehydes derived from lipid peroxidation, methylating agents, DNA hydrolysis, hydrolytic deamination and carbonyl stress [35]. Endogenous damage may also arise due to genotoxic stress from cellular processes such as transcription and replication that overwhelm high-fidelity of DNA repair in an otherwise repair-competent background [36,37]. Majority of these lesions (approximately 75%) are single-strand DNA breaks (SSBs), which can be converted to DNA double-strand breaks (DSBs) when replication forks come across SSBs. Although less frequent, DSBs are far more dangerous and difficult to repair as they represent a complete physical break in the DNA backbone [1,2].

Since chromatin is the physiological template for DNA repair machinery, the structural context of chromatin environment may facilitate or alternatively make it more challenging for repair factors to maintain and restore genome integrity [4,38]. Depending on the lesion, cells have acquired specialized DNA repair pathways that ensure genome stability. In this regards, base mismatches during replication are corrected by mismatch repair (MMR), while small chemical modifications of DNA bases are repaired by base excision repair (BER) through excision of the damaged base [34,39]. More complex DNA adducts, such as pyrimidine dimers and intra-strand crosslinks, are repaired by nucleotide excision repair (NER), while inter-strand crosslinks (ICLs) are repaired through cooperation of several repair pathways including NER [40,41]. SSBs are repaired by single-strand break repair (SSBR), whereas DSBs are processed either by error prone non-homologous end joining (NHEJ) or error free homologous recombination (HR) pathways depending upon cellular context including cell cycle phase [42,43].

Notably, the arrangement of the genome into heterochromatin- and euchromatin-like domains may substantially influence regional variations in genome instability in human somatic cells [44]. This variation in genome instability was found to be particularly predominant in cancer cells where the major determinant of regional mutation rate is chromatin structural organization, with DNA base substitutions elevated in heterochromatic regions and late replicating domains while less frequent in more open, early replicating chromatin [44,45]. Accordingly, higher frequency of somatic mutations (most likely due to base substitutions) in cancer genomes positively correlates with levels of heterochromatic histone PTMs such as H3K9me3/2 and H4K20me3 and negatively correlates with genetic and epigenetic features associated with open chromatin, such as histone H3 and H4 N-terminal tail acetylation, H3K4/K36me3, GC content of DNA, and highly expressing genes [44]. This indicates that DNA lesion formation and efficiency of repair can be influenced by chromatin structure landscape. Several factors are likely involved including differential accessibility of heterochromatic regions to DNA repair machineries, variation in the ability to signal repair or perhaps increased exposure to mutagens at the nuclear periphery rather than sequence specific differences in mutation rates [44,45,46,47]. Accordingly, it was shown that MMR is more efficient in euchromatic early replicating regions of the genome, suppressing the accumulation of mutations in these regions [47]. 

In fact, numerous studies over the years lend support to a central role for chromatin plasticity in DNA repair, where repair of lesions follows a set of orchestrated events aptly described as the “access-repair-restore” model [48]. Evidence for this model originally emerged from studies measuring DNA accessibility in response to UV-induced damage [49]. Herein, the initial response to DNA damage includes rapid decompaction of surrounding chromatin to allow efficient detection of the damage as well as enable access for repair proteins [50,51]. In line with this, heterochromatin is refractory to initial signaling events in DDR and γH2A.X, one of the earliest signs of DNA damage [3], is mostly limited to the periphery of heterochromatic regions [6,52]. Furthermore, decreasing the degree of chromatin compaction by inhibiting histone deacetylases (HDACs), which functions by removing the acetyl groups from nucleosomal histones and thereby keeping the chromatin more compact, or by reducing the levels of the linker histone H1, enhances DDR signaling and the extent of γH2A.X spreading from the lesion [7,53]. However, the simple model that open and relaxed chromatin favors local DDR signaling is probably an oversimplification because euchromatic regions such as gene promoters that are occupied by transcription machinery also constitute a barrier for repair machinery and γH2A.X spreading [54]. A rather interesting observation in this regard is that nucleosome free regions (NFR), if bound by transcription factors, have very high mutation rates by blocking accessibility of nucleotide excision repair machinery as compared to neighboring NFR not bound by any protein [55,56,57]. Furthermore, increased nucleosome occupancy coincides with increased mutation rates [55], whereas higher transcription rates generally correlate with lower prevalence of mutations on transcribed genes even if transcribed genes are located in or near heterochromatic regions [58,59]. This suggests that the structural constraints imposed by tightly packed chromatin on DNA repair machinery are alleviated by gene expression. Furthermore, consistent with differences in repair across the genome, when either the NER or MMR pathway is lost, mutations are more evenly distributed [45,47].

## 5. Chromatin Structure Regulates Transcription and Replication to Maintain Genome Stability

Since the initial discovery of chromatin structure as a repeating array of nucleosomes, it has been speculated that it plays much more diverse roles than mere packaging and compaction of genomic DNA. This is substantiated by observations that presence of nucleosomes blocks transcription in-vitro [60] as well as deletions of histone N-terminal tails leading to changes in gene expression [61,62]. The structural and physical organization of chromatin is therefore an important functional determinant of DNA-based processes including transcription, recombination, DNA repair, replication, kinetochore and centromere formation etc. In this regard, histones PTMs and assembly of histone variants play a fundamental role in creating chromatin functional domains by directly modulating chromatin structure, in turn regulating the DNA processing activities [63]. Any perturbation in establishing histone PTMs and histone variants-based chromatin environments is reflected into defects in chromatin structural organization (see Table 1) and may directly affect the smooth working of DNA-based transactions and manifest as endogenously arising DNA damage that promotes genome stability.

DNA replication is highly coordinated with other DNA-based processes occurring concurrently on chromatin, such as transcription, to ensure faithful duplication of both genetic and epigenetic features and to safeguard genomic stability. Given that human cells have to duplicate 6 × 10^9^ nucleotides in every cell cycle, replicating DNA with high fidelity is a challenging task and prone to errors and disruptions. Consequently, cells have evolved to instruct the DNA replication program in spatially and temporally regulated manner [64], which is, in part, determined by the chromatin structural landscape [65]. In-vitro chromatin replication assays show that presence of nucleosomes enforces DNA replication origin specificity by preventing non-specific MCM (Minichromosome maintenance) helicase loading, while helicase activation and replisome progression requires chromatin structure modulation by chromatin remodeling complexes [66,67]. In line with these observations, recent unpublished data from our group indicates that histone H4K20 methylation dependent chromatin compaction determines the origin specificity in late mitosis and early G1 phase of the cell cycle. Loss of control of chromatin compaction leads to aberrant loading of replication licensing factors and uncontrolled replication initiation, which results in loss of genome integrity.

The highly regulated spatio-temporal program of DNA replication also ensures a sequential replication of different regions of genome, some replicated earlier than others during S phase [68]. In this regard, early and late replicating regions or domains correlate, respectively, with open and closed three-dimensional chromatin compartments, as identified by high-resolution chromosome conformation capture (Hi-C) [69]. It has been shown that these replication domains are already established during G1 phase and coincide with the establishment of higher order chromatin structure after mitosis [70]. The deregulation of replication timing program by changes in chromatin domain organization may lead to a disruption in the spatio-temporal segregation of early and late replicating domains [71]. In this regard it will be interesting to fully understand if changes in replication timing affects the accurate completion of replication in S-phase, which is critical to avoid entering mitosis with unrepaired DNA that leads directly to loss of genome integrity [72]. 

Chromatin structure imposes significant obstacles on all aspects of transcription that are facilitated by RNA polymerase II. However, the plasticity and dynamic nature of chromatin structure provides suitable environment and landing platforms for regulatory proteins, which in turn regulate gene expression. Multiple mechanisms ensure strict regulation of chromatin dynamics during transcription including histone modifications, chromatin remodeling, histone variant incorporation, and histone turnover. Moreover, chromatin signatures around regulatory DNA sequences promote proper transcription and prevent transcription related genome instability. Transcription occurs in three well defined steps, initiation, elongation and termination, all of which requires chromatin structural modulation [73]. It is believed that chromatin remodeling complexes and histone chaperones work together to advance transcription through chromatin template and help redeposit histones back onto transcribed regions [74]. Any disruption in histone redeposition would leave exposed free DNA and allow transcription factors to bind to cryptic promoters and initiate transcription from internal start sites [75]. This leads to excessive pervasive transcription and excessive accumulation of DNA–RNA hybrids in the form of structures called R-loops that eventually block transcription and replication, potentially resulting in replicative stress and genome stability [76]. The DNA–RNA hybrids invariably expose single stranded DNA (ssDNA) which is more unstable and susceptible to DNA breaks and genomic instability by nucleases, activation-induced cytidine deaminase and exogenous genotoxic stress [77]. It has also been suggested that the ssDNA resulting from R-loop formation is more susceptible to spontaneous deamination of deoxy-Cytidine to deoxy-Uridine, leading to SSBs, DSBs and recombination [76,78,79]. Furthermore, the redeposition and deacetylation of histones after transcription termination are both required to maintain chromatin in a stable conformation within the genic regions and help maintain overall genome integrity. However, it is not known if DNA exposure due to failure of histone deposition after transcription termination may allow for DNA endonuclease attack and DNA breaks in the genic regions.

Given that DNA replication and transcription machineries share a common template, there is evidence indicating their collision with each other either co-directionally or head-on leads to genome instability [80]. Furthermore, highly transcribing genes, especially during S phase have significantly increased probability of conflicts with the replication fork and this eventually poses a substantial threat to genome integrity [81,82]. Accordingly, collisions between DNA and RNA polymerases present a high risk of chromosomal rearrangements including deletions, duplications, inversions, and translocation events and may lead to development of cancers [82]. Interestingly, these chromosomal rearrangements are more frequent in transcriptionally active early replicating genomic regions as compared to more compact heterochromatic regions [83]. The underlying reason may be that about two thirds of the transcribing genes are located in the early replicating, DNase I sensitive euchromatin [84], and therefore, more prone to replication-transcription collisions as compared to gene poor and transcriptionally dormant heterochromatin. However, it is generally believed that euchromatic DSBs are more rapidly repaired owing to increased signaling and better access to repair machineries as compared to more compact heterochromatin [85]. Therefore, it is likely that in early replicating regions increased DNA damage rather than deficient DNA repair might underlie the predominance of rearrangements.

## 6. Chromatin Structure Regulates DNA Damage Responses

The roles and regulation of chromatin structure in the response to exogenous DNA damage is better understood than its roles in suppressing endogenously arising DNA damage and genome instability. Thus, the aforementioned “access-repair-restore” model has gained support as an explanation for local chromatin changes upon DNA damage, particularly after UV radiation exposure [48,49]. In addition to local decompaction, localized UV-irradiation has also been shown to result in a global chromatin relaxation response affected by p53 [50,51,86]. The authors here suggested that a local lesion site is detected in a transcription-associated manner leading to p53-mediated global chromatin relaxation response facilitating detection of further lesions within the genome. Studies using a photoactivatable version of GFP-tagged histone H2B showed that chromatin also undergoes an ATP-dependent local expansion immediately after DNA damage [87]. This localized expansion corresponds to a 30–40% reduction in chromatin fiber density in the vicinity of DSBs and occurs independently of H2A.X and ATM, the two early effectors of DDR. Chromatin relaxation may be affected through alterations in histone PTMs, mobility of chromatin binding proteins, or through disruption of whole nucleosomes as described below [88].

Chromatin decompaction and increased fiber flexibility was recently reported to be accompanied by histone degradation in response to DNA damage [89]. In this regard, the nucleosome remodeler INO80 is required for a proteasome-mediated 20–40% drop in histone levels. Using a system to create DSBs at defined sites in the human genome, Berkovich et al. showed that ATM and NBS1 are required for nucleosome disruption following DNA damage [90]. Nucleosome eviction can extend over a region of ~3 kb or less on each side of a DSB and efficient histone loss is required for repair. Nucleolin, a protein with histone chaperone activity, is recruited to the DSBs through interaction with RAD50 of the MRN complex where it directly binds and removes H2A/H2B dimer leading to a partial nucleosome disruption in G1 phase of the cell cycle [91]. A complete nascent nucleosome disruption is seen in the asynchronous population of cycling cells where additional eviction of H3/H4 dimer is dependent on the initial H2A/H2B removal by nucleolin and human isoforms A and B of the histone chaperone anti-silencing function 1 (ASF1) seem to be required for this function. In contrast to the restricted disruption of only H2A/H2B dimer in G1-arrested cells associated with NHEJ repair, complete nucleosome disruption occurs in cycling cells (during S and G2 phases), which is associated with DNA end resection by C-terminal binding protein interacting protein (CtIP) endonuclease, a process that directs DSB repair (DSBR) to the HR pathway [91]. Chromatin remodeling through nucleosome disruption is important for efficient DSBR as down regulation of nucleolin impedes recruitment of repair factors resulting in inefficient repair.

**Figure 2:**
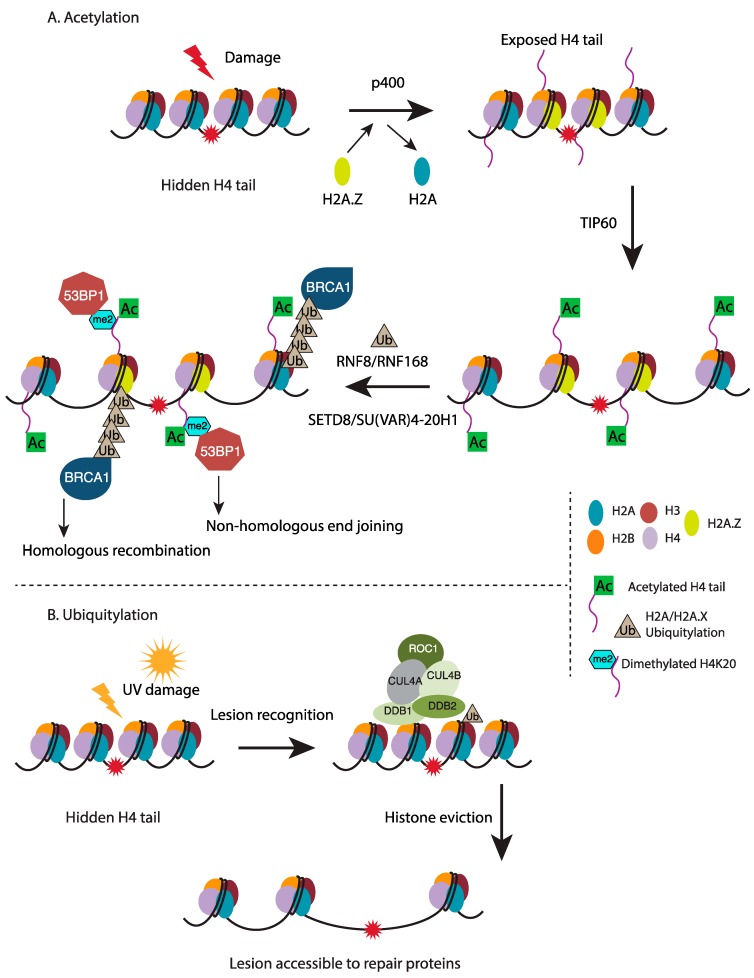
Chromatin structure is modified in response to DNA damage: (**A**) Acetylation: Following irradiation, p400 facilitates exchange of the histone variant H2A.Z in place of histone H2A to destabilize nucleosomes surrounding the DSB in turn exposing the histone H4 tail that can now be acetylated by the TIP60 histone acetyltransferase. H4 hyperacetylation further relaxes the chromatin structure and enables access to downstream repair factors. RNF8/RNF168-dependent histone ubiquitylation provides docking site for the HR-promoting BRCA1 complex. Alternatively, histone H4 lysine 20 dimethylation (affected by SU(VAR)4-20H1 that uses H4K20me1, catalyzed by SETD8, as substrate) is recognized by the NHEJ factor, 53BP1; (**B**) Ubiquitylation: Following UV-induced damage, NER lesion recognition elements are recruited to sites of damage where they function to facilitate ubiquitylation of histones H3 and H4 by the CUL4-DDB-ROC1 ubiquitin E3 ligase, leading to their eviction from the nucleosome. This release of histones H3 and H4 from nucleosomes at damage site leads to increased accessibility for downstream NER factors.

In addition to nucleosome disruption, extensive chromatin decompaction as evidenced by decreased intensity of chromatin labeling, increased H4K5 acetylation and decreased H3K9 dimethylation, observed at DSB sites as early as 15 min after irradiation [92]. Histone H4 hyperacetylation by the histone acetyltransferase (HAT) TIP60 and HAT cofactor TRRAP (Transformation/Transcription Domain Associated Protein) at sites of DSB facilitates accumulation of repair proteins [93]. In addition to histone acetylation, TIP60 and TRRAP also associate with p400, a SWI/SNF DNA-dependent ATPase, to destabilize nucleosomes in response to DSBs in an ATM-independent manner [94]. Instead, p400 recruitment to DSBs is dependent on MDC1 (Mediator of DNA damage checkpoint protein 1) interaction with γH2A.X and is required for exchange of histone H2A.Z onto nucleosomes at DSB site (Figure 2A) [95]. H2A.Z exchange leads to a more relaxed chromatin state through enhanced acetylation by TIP60 and is required for RNF8/RNF168-dependent ubiquitylation of chromatin adjacent to DSBs that in itself serves as a docking site for repair complexes including BRCA1 that promotes DSBR through HR [96]. RNF168 also directly interacts with the HR-promoting factor PALB2 (Partner and localizer of BRCA2) to couple HR to RNF168 mediated H2A ubiquitination in S/G2 phases of the cell cycle [97]. Additionally, H4 hyperacetylation and chromatin ubiquitylation facilitate loading of NHEJ-associated 53BP1 (p53-binding protein 1) to chromatin by revealing H4K20me2 residues, a histone mark that is recognized by the tudor domain of 53BP1 [98]. Similar to the exchange of H2A.Z, the chromatin remodeler CHD2 (Chromodomain-helicase-DNA-binding protein 2) has also been shown to trigger rapid chromatin expansion through incorporation of histone H3 variant H3.3 at sites of DNA damage [99]. H3.3 deposition at DSBs by CHD2 is dependent on poly (ADP-ribose) polymerase 1 (PARP1) and promotes the assembly of NHEJ complexes for efficient repair of DSBs.

Additionally, histone ubiquitylation has also been shown to contribute to chromatin structural changes within other repair pathways. Upon UV irradiation, histones H3 and H4 are ubiquitylated by the CUL4-DDB-ROC1 ubiquitin E3 ligase leading to their release from nucleosomes due to altered stability (Figure 2B) [100]. In this manner, ubiquitylation of histones H3 and H4 serves in making the UV lesion more accessible to repair proteins in turn facilitating repair as CUL4 depletion results in impaired cellular response to UV-induced damage. Whereas acetylation and ubiquitylation promote an open chromatin structure, the role of histone methylation in response to DNA damage is more complex. Some evidence suggests a decrease in methylation levels favor a more relaxed chromatin state. SETD8, a histone methyltransferase required for monomethylation of H4 lysine 20 residues is degraded following exposure to DNA damaging agents such as UV and IR [101,102]. Monomethylation of H4K20 is required for di- and tri-methylation of this residue by the SU(VAR)4-20H1 and SU(VAR)4-20H2 enzymes [103,104], respectively and thus reduced H4K20me1 levels could probably help modulate chromatin structure following DNA damage through reduced levels of H4K20me3. Further work on defining the role of histone methylation in modulating chromatin structure in the context of repair processes is needed.

In addition to DNA damage induced histone PTMs, the chromatin landscape also changes through mobilization of bound proteins to make the DNA strands more accessible to repair factors. For instance, HP1-β, a member of the HP1 family, seen bound to H3K9me is mobilized in response to DNA breaks. The interaction between HP1-β and H3K9me is transiently disrupted upon phosphorylation by casein kinase 2 (CK2), an enzyme implicated in DNA damage sensing and repair [105]. Thus, disruption of nucleosomes on a whole as well as alterations in histone stability, histone PTMs, and proteins that associate with specific histone PTMs contribute to modulating the chromatin structure in response to DNA damage in a manner that facilitates repair enzyme access.

## 7. DNA Repair in the Context of Heterochromatin

The influence of chromatin compaction on the induction of DNA breaks after ionizing radiation has been studied both in-vivo and in-vitro. The organization of genomic DNA into a chromatin fiber both at the very basic nucleosomal level and also the higher-order chromatin structures provide significant protection against break induction [8,106,107]. Furthermore, decompacted chromatin is more susceptible to DNA damage caused by chemical agents, such as cisplatin [8]. Studies in Drosophila suggest that compaction of chromatin into a more heterochromatin-like state is favorable for survival upon HU-induced damage [108]. Chromatin compaction seen in heterochromatin protects DNA from damage but is also thought to be refractory to repair if a lesion does occur, as seen by its ability to block the expansion of H2A.X phosphorylation [109]. Studies in both yeast and mammalian cells have revealed that γ-H2A.X foci at DSBs form at lower levels in heterochromatin as compared to euchromatin [6,7]. Mutations in stonewall (stwl), a heterochromatin-associated protein, are associated with decreased levels of tri-methylated H3K27 and H3K9 (two hallmarks of silent chromatin) and the euchromatin-like state in this context might be more vulnerable to damage. This protective effect of chromatin compaction may well be due the presence of large number of non-histone chromatin proteins that physically shield the genomic DNA [110].

**Figure 3:**
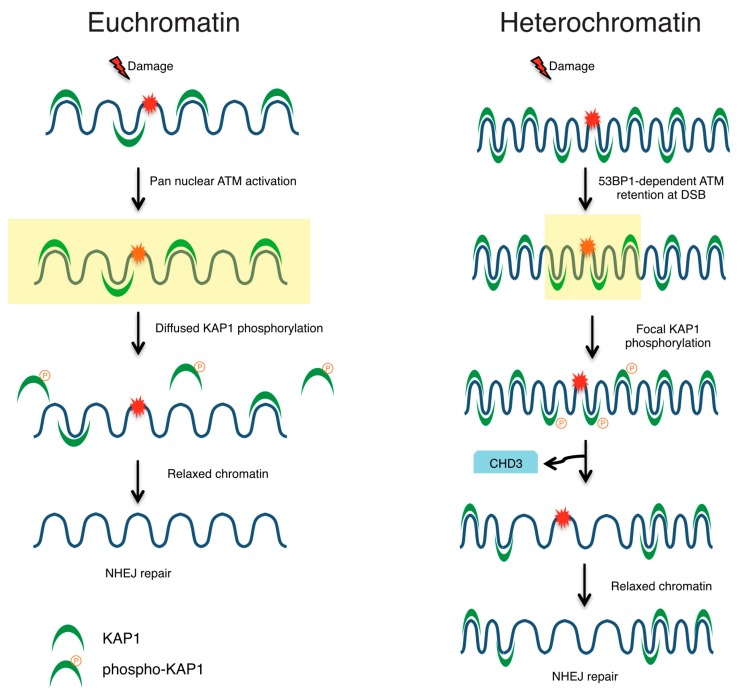
DNA damage response in euchromatin vs. heterochromatin. DNA DSBs within euchromatic regions of the genome result in global activation of the ATM kinase that in turn affects KAP1 phosphorylation in a diffused manner. KAP1 phosphorylation and release from chromatin promotes repair through enhanced access to damage site. However, when damage occurs within heterochromatic regions, ATM activity is retained at the site of DSB in a 53BP1-dependent manner. In this manner, 53BP1 functions to retain several factors onto the damage site and also facilitate localized KAP1 phosphorylation and dissociation of CHD3 in turn leading to chromatin relaxation.

Within euchromatin, a complex of the heterochromatin-associated proteins KAP1 (KRAB-associated protein 1) and HP1 together with the H3K9 methyltransferase SUV39H1 is loaded onto DSBs rapidly following damage where it facilitates H3K9 tri-methylation [111]. This in turn promotes loading of additional KAP1/HP1/SU(VAR)3-9H1 complexes through HP1 binding to H3K9me3 thereby spreading the binding to tens of kilobases from the lesion site. DNA damage induced Tyr44 phosphorylation of TIP60 promotes its binding to the H3K9me3 domains leading to ATM and histone H4 acetylation [112]. ATM acetylation enhances its activation and leads to KAP1 phosphorylation (pKAP1) and release of the complex from chromatin removing the repressive mark. It could be that H3K9 tri-methylation represents a transient shift to repressive chromatin that is required for TIP60 recruitment and ATM activation in more open regions of the chromatin. However, DSBs within heterochromatin undergo localized phosphorylation of KAP1 leading to retention of the protein into foci at sites of damage, a process that is dependent on the ATM effector protein, 53BP1 [85]. MRE11-NBS1 accumulation at late-repairing DSBs is augmented by 53BP1 in order to accrue active ATM for enhanced localized pKAP1. KAP1 phosphorylation directly perturbs its interaction with the nucleosome remodeler, CHD3 (Chromodomain-helicase-DNA-binding protein 3) thereby dispersing CHD3 from heterochromatin and enabling relaxation for efficient repair [113]. Thus, ionizing radiation induced foci (IRIF) spatially concentrate ATM activity in regions otherwise inhibitory to repair to promote localized alterations for efficient DSBR. Hence, the initiation of the DDR signaling cascade seems to vary depending on whether the lesion occurs within euchromatin or heterochromatic regions of the genome (Figure 3). DDR within more compacted regions may likely be delayed in an effort to concentrate effector proteins to the damage site [85]. Contrary to this notion, Janssen et al. observed similar kinetics for DSBR in euchromatin and heterochromatin using an in vivo system in Drosophila [114]. They used live cell imaging to study repair kinetics at a single DSB site in both heterochromatic and euchromatic loci and suggested that cells can employ either NHEJ or HR for repair of heterochromatic DSBs that are resolved with similar kinetics as those that occur within euchromatic regions of the genome. This observation could however be due to the different types of DSB ends studied previously compared to those studied here. Additionally, the Drosophila chromatin landscape and genome volume varies widely from that of mammalian cells and could also explain the conflicting results. In the future, application of a similar single break system in mammalian cells could help better address the kinetics and regulation of repair response in euchromatin vs. heterochromatin.

## 8. Chromatin Structure Influences Repair Pathway Choice

The choice between either a homology-directed repair (HR) pathway or non-homologous end joining (NHEJ) for DSBR is dependent on several factors including cell cycle status wherein HR is active in the S/G2 phases; and NHEJ is the single DSBR pathway active in all cell cycle phases [115]. Additionally, functionally opposed chromatin alterations have also been linked to pathway choice during DSBR, where presumably gene rich transcriptionally active euchromatic regions favor error free HR over more error prone NHEJ pathway. Chromatin compaction restricts mobility and thus, could be inhibitory to homology search during HR and is thought to in turn promote repair of DSBs through NHEJ where the broken ends could be simply re-ligated [116]. Indeed, chromatin decompaction resulting from INO80-mediated histone degradation and generalized reduction in nucleosome occupancy leads to enhanced recombination rates following DNA damage through increased chromatin mobility [89]. In this regard, SETD2 methyltransferase mediated H3K36 trimethylation, a histone mark associated with active transcription has been shown to be required for the recruitment of CtIP (CtBP-interacting protein) in turn promoting DNA end resection and HR [117]. Low levels of H3K36me3 are thus associated with reduced HR repair and significantly increased deletions arising as a result of end-joining events. In addition to this, histone acetylation that mediates chromatin decompaction can also promote HR [118]. For instance, while the TRRAP/TIP60/p400 complex mediated exchange of H2A.Z onto nucleosomes restricts DNA resection and is required for loading of the Ku proteins favoring NHEJ [95], a separate study showed that p400 facilitates homologous recombination (HR) at DSBs through RAD51 recruitment [119]. In addition to this, other studies also support a role for the TRRAP/TIP60/p400 complex in promoting HR [93,120]. Further, depletion of TRRAP impaired DNA repair by HR due to reduced DNA damage induced H4 hyperacetylation that could be counteracted by chromatin relaxation using hypotonic conditions [93]. Similarly, TIP60 deficiency also reduced BRCA1 recruitment at DSB with a corresponding increase in 53BP1 [120]. This HR-promoting role for the complex is indeed through its histone acetylation function as inhibition of the histone deacetylase (HDAC) enzyme reduced HR. In part, the NHEJ inhibitory role of TIP60 H4 acetylation could be through blocking of 53BP1 binding to the H4K20me2 mark. Additional studies also argue that chromatin relaxation facilitates HR and not NHEJ. For instance, ATM induced local chromatin decompaction is thought to be required for mobility of DSB containing chromatin domains, mainly in search of homology partners to affect HR [121]. However, this expansion is regulated in a manner dependent on cell cycle status and chromatin context as excessive mobility could also lead to chromosomal translocations through erroneous recombination events.

One of the factors that affect regulated chromatin expansion and mobility is the nuclear oncogene SET that is recruited to DSBs to limit uncontrolled DNA end resection and HR [122]. SET interacts with KAP1 and promotes the retention of KAP1 and HP1 on chromatin in turn compacting the chromatin and restricting repair through HR. Additionally, two components of repressive chromatin structure, macro-histone variant macroH2A1 and the H3K9 methyltransferase PRDM2 have been shown to direct repair of DSBs into the BRCA1-dependent HR pathway [51]. An ATM-dependent accumulation of macroH2A1 and PRDM2 (PR domain-containing protein 2) at lesion sites promotes H3K9 dimethylation in the flanking regions to modulate shift from accessible to compacted chromatin in turn promoting CtIP-dependent end resection. Differential local concentrations of HP1 could also play a critical role in DSBR pathway choice, wherein HP1 has been shown to stimulate HR by promoting DNA-end resection [123]. In G2/M, chromatin abundant for HP1 could be repaired through BRCA1-dependent HR, while HP1-depleted cells show increased 53BP1 association and are dependent on NHEJ for repair. Interestingly, while HP1-α and HP1-β stimulate HR by promoting DNA-end resection, the HP1-γ subtype, which is mostly enriched in euchromatin, has an inhibitory role [124]. Additional evidence to support HR dominance in DSBR within compacted chromatin comes from the finding that the heterochromatin associated factor KAP1 also favors repair of DSBs by HR [125]. KAP1 depletion or ATM-mediated KAP1 inactivation through phosphorylation leads to chromatin relaxation that allows repair of resected DSBs by NHEJ. 

Thus, chromatin relaxation can promote both HR and NHEJ, which could be cell cycle or genome location specific. However, a contributing aspect could also be limited availability of techniques to accurately measure NHEJ frequency within the context of chromatin. It is challenging to assess repair through the NHEJ pathway as the resulting ligated DNA strands if repaired precisely are not detectable in most assays, hence, several cycles of cleavage and repair may be missed leading to an erroneous estimate of NHEJ vs. HR events [126]. To study the balance between HR and NHEJ in the context of relaxed or compact chromatin status, it is important to apply complementary methods to measure both types of repair at the same time using a defined chromosomal site.

## 9. Chromatin Decompaction Activates and Determines DDR Intensity

Further to providing access to lesion sites, changes in chromatin structure also play a role in DDR through initiating a DNA damage-signaling cascade. In this regard, it has been suggested that it is in fact a change in the chromatin structure resulting from IR-induced DNA strand breaks that serves as an initiating signal to activate ATM [127]. Moreover, ATM can be activated even in the absence of detectable DNA breaks by chromatin decompaction through hypotonic swelling or by treatment with the HDAC inhibitor trichostatin A (TSA). However, when ATM is activated in this manner, localized ATM substrates at the site of breaks (for example, H2A.X) fail to be phosphorylated, whereas substrates present elsewhere in the nucleus (for example, p53) can still be phosphorylated. This could be to help the cell respond appropriately to local damage vs. global genomic stress.

Reduction in the expression levels of linker histone H1 is associated with a more “open” chromatin structure in embryonic stem (ES) cells [128] and this has been shown to render them hyperresistant to DNA damage in part due to enhanced DDR at the lesion site [53]. Additionally, these cells also exhibit hypersensitive checkpoints as a result of increased ATR (Ataxia telangiectasia and Rad3-related protein) signaling at each DNA break. This enhanced DDR intensity resulting from a more relaxed chromatin structure was mirrored in cells treated with TSA arguing to the fact that chromatin decompaction not only initiates DDR signaling but also boosts intensity of the response. This relationship between chromatin and the DDR could be crucial in deciphering the heterogeneity of cellular responses to DNA damage in vivo. For instance, CD133+ glioma cancer stem cells show a more robust DDR activation that in turn promotes radioresistance in the tumor-forming population [129]. Since, increased overall chromatin accessibility is an innate property of stem cells that is lost on differentiation [130], an exciting avenue for future research could be to therapeutically exploit chromatin compaction in order to radiosensitize cancer stem cells.

### Compaction Can Activate DDR

Whereas, chromatin decompaction can initiate DDR signaling, Burgess et al. suggested that forced chromatin compaction also induced DDR signaling albeit only the upstream ATM- and ATR-dependent factors were activated in a break-independent manner [131]. However, the studies were carried out in defined chromatin domains using a protein-chromatin tethering system with ~10 kb tandem arrays of tightly bound Lac operator (lacO) DNA [132]. The ATM activation observed could be influenced by the replication stress generated by such an array. Indeed, hypoxia-induced replication stress has also been shown to activate ATM in the absence of DNA damage through H3K9 tri-methylation, a histone modification associated with heterochromatin [133]. However, prolonged chromatin compaction is refractory to downstream signaling and impairs survival after DNA damage [131]. Indeed, work in our lab has also shown that the presence of chromatin in itself is inhibitory to ATM activation as seen through reduced CHK2, KAP1 and ATM S198 phosphorylation (unpublished data). Dynamic changes in chromatin compaction status may thus be required to first signal for repair and then allow access to the repair machinery.

## 10. Perspectives and Open Questions

The plasticity of chromatin structure enables access to cellular machineries to perform DNA-based processes as well as protects the underlying sequence information contained in the genome. For instance, deregulation of the replication-timing program by changes in chromatin domain organization may lead to a disruption in segregation of the early and late replicating domains. However, it is not known if changes in replication timing program lead to loss of genome integrity. Therefore, it would be very interesting to study the effect of chromatin structural organization in this context.

The increased DNA DSBs and erroneous recombination events arise in repetitive DNA sequences primarily due to their transcriptional and transpositional activities, giving rise to genome instability. Therefore, heterochromatinization of repetitive elements appears as an evolutionarily conserved mechanism to prevent unnecessary transcription and transposition of these elements in the genome and in turn safeguard genome stability. In this manner, chromatin structural organization functions to repress genomic instability arising from unstable elements within the genome by imposing structural restraints to prevent their activation. However, the transcriptionally dynamic nature of repetitive elements begs the question if all repeats are the same in terms of their capacity to cause genome instability. 

The complexity and diversity in chromatin organization as evident by the presence of “secondary” and “tertiary” chromatin structures may also pose a significant obstacle to repair of DNA lesions. Importantly, endogenous damage leads to higher number of mutations in more compact chromatin structure mainly due to restricted accessibility and DDR signaling. Thus, the dynamic nature of chromatin compaction is crucial to genome integrity through structurally restricting unstable repetitive elements on the one hand while allowing access to repair factors upon damage on the other hand. In this regard, a thorough mechanistic understanding of factors and modifications involved in altering chromatin structure in response to both endogenous and exogenous damage is still lacking.

Studies so far argue that chromatin structure also influences the repair choice for DSBR and thus it would be interesting to test whether cancers deficient in one of the repair pathways could be targeted using a synthetic lethality approach, whereby the choice between HR or NHEJ is forced through chromatin structure alteration. Finally, it is generally thought that more open chromatin structure promotes repair efficiency, however, transcriptionally active open chromatin regions are also prone to chromosomal rearrangements as compared to more compact, transcriptionally inactive heterochromatic regions. Moreover, altering chromatin structure would also deregulate other DNA-templated processes such as transcription and replication, which could lead to genomic catastrophe. Hence, further studies are vital to obtain a deeper understanding of chromatin-based regulation of DNA processes in order to ascertain the role of chromatin structural organization in maintaining genome stability.

## Figures and Tables

**Table 1:** List of histone PTMs implicated in DDR and chromatin structure modulation.

Histone	Residue	Modification	Enzyme	Effect on Chromatin Compaction	Proposed Cellular Function
H2A	Ser139	phosphorylation	ATM, ATR, DNA-PKcs	not known	DNA repair
	Lys119	ubiquitylation	RING2, BRCA1	compaction	spermatogenesis
H3	Lys4	methylation	SETD7/9, MLL	not known	permissive euchromatin (di-Me), transcriptional activation
	Lys9	acetylation	GCN5, SRC1, unidentified	decompaction	transcriptional activation, histone deposition
		methylation	SU(VAR)3-9H1, Clr4, EHMT2, SETDB1	compaction	transcriptional silencing (tri-Me), transcriptional repression, genomic imprinting, DNA methylation (tri-Me), transcriptional activation
	Lys27	methylation	EZH2, EHMT2	compaction	transcriptional silencing, X inactivation (tri-Me)
	Lys36	methylation	SETD2	decompaction	transcriptional activation (elongation)
H4	Lys5	acetylation	HAT1, TIP60, ATF2, HPA2, p300 HAT	decompaction	histone deposition, transcriptional activation, DNA repair
	Lys8	acetylation	GCN5, PCAF, TIP60, ATF2, Elp3, p300 HAT	decompaction	transcriptional activation, DNA repair
	Lys12	acetylation	HAT1, TIP60, HPA2, p300 HAT	decompaction	histone deposition, telomeric silencing, transcriptional activation, DNA repair
	Lys16	acetylation	GCN5, TIP60, ATF2, Sas2	decompaction	transcriptional activation, DNA repair, euchromatin
	Lys20	methylation	SETD8, SU(VAR)4-20H1, SU(VAR)4-20H2	compaction	heterochromatin (tri-Me), transcriptional activation/silencing, checkpoint response, 53BP1 loading following DSBs (di-Me)

ATM; ataxia-telangiectasia mutated, ATR; ataxia telangiectasia and Rad3-related protein, DNA-PKcs; DNA-dependent protein kinase catalytic subunit, SET7/9; SET domain-containing protein 7, MLL; Mixed-lineage leukemia protein 1, GCN5; General control of amino acid synthesis protein 5-like 2, SRC1; Steroid receptor coactivator 1, SU(VAR)3-9H1; Suppressor of Variegation 3-9 Homolog 1, Clr4; Cryptic loci regulator 4, EHMT2; Euchromatic histone-lysine *N*-methyltransferase 2, SETDB1; SET domain bifurcated 1, EZH2; Enhancer of zeste homolog 2, SETD2; SET domain-containing protein 2, HAT1; Histone acetyltransferase 1, TIP60; 60 kDa Tat-interactive protein, ATF2; Activating transcription factor 2, HPA2; Inactive heparanase-2, p300 HAT; Histone acetyltransferase p300, PCAF; P300/CBP-associated factor, Elp3; Elongator complex protein 3, Sas2; Something about silencing protein 2, SETD8; SET domain-containing protein 8, SU(VAR)4-20H1; Suppressor of Variegation 4-20 Homolog 2, SU(VAR)4-20H2; Suppressor of Variegation 4-20 Homolog 2.

## References

[1] Jackson S.P., Bartek J. (2009). The DNA-damage response in human biology and disease. Nature.

[2] Ciccia A., Elledge S.J. (2010). The DNA damage response: Making it safe to play with knives. Mol. Cell.

[3] Rogakou E.P., Pilch D.R., Orr A.H., Ivanova V.S., Bonner W.M. (1998). DNA double-stranded breaks induce histone H2AX phosphorylation on serine 139. J. Biol. Chem..

[4] Soria G., Polo S.E., Almouzni G. (2012). Prime, repair, restore: The active role of chromatin in the DNA damage response. Mol. Cell.

[5] Papamichos-Chronakis M., Peterson C.L. (2013). Chromatin and the genome integrity network. Nat. Rev. Genet..

[6] Cowell I.G., Sunter N.J., Singh P.B., Austin C.A., Durkacz B.W., Tilby M.J. (2007). GammaH2AX foci form preferentially in euchromatin after ionising-radiation. PLoS ONE.

[7] Kim J.A., Kruhlak M., Dotiwala F., Nussenzweig A., Haber J.E. (2007). Heterochromatin is refractory to gamma-H2AX modification in yeast and mammals. J. Cell Biol..

[8] Takata H., Hanafusa T., Mori T., Shimura M., Iida Y., Ishikawa K., Yoshikawa K., Yoshikawa Y., Maeshima K. (2013). Chromatin compaction protects genomic DNA from radiation damage. PLoS ONE.

[9] Luger K., Dechassa M.L., Tremethick D.J. (2012). New insights into nucleosome and chromatin structure: An ordered state or a disordered affair?. Nat. Rev. Mol. Cell Biol..

[10] Tremethick D.J. (2007). Higher-order structures of chromatin: The elusive 30 nm fiber. Cell.

[11] Ricci M.A., Manzo C., García-Parajo M.F., Lakadamyali M., Cosma M.P. (2015). Chromatin fibers are formed by heterogeneous groups of nucleosomes in vivo. Cell.

[12] Woodcock C.L., Ghosh R.P. (2010). Chromatin higher-order structure and dynamics. Cold Spring Harb. Perspect. Biol..

[13] Allis C.D., Jenuwein T. (2016). The molecular hallmarks of epigenetic control. Nat. Rev. Genet..

[14] Trojer P., Reinberg D. (2007). Facultative heterochromatin: Is there a distinctive molecular signature?. Mol. Cell.

[15] Khorasanizadeh S. (2004). The nucleosome: From genomic organization to genomic regulation. Cell.

[16] Peng J.C., Karpen G.H. (2008). Epigenetic regulation of heterochromatic DNA stability. Curr. Opin. Genet. Dev..

[17] Padeken J., Zeller P., Gasser S.M. (2015). Repeat DNA in genome organization and stability. Curr. Opin. Genet. Dev..

[18] Vader G., Blitzblau H.G., Tame M.A., Falk J.E., Curtin L., Hochwagen A. (2011). Protection of repetitive DNA borders from self-induced meiotic instability. Nature.

[19] Yang J., Li F. (2017). Are all repeats created equal? Understanding DNA repeats at an individual level. Curr. Genet..

[20] Henikoff S. (2000). Heterochromatin function in complex genomes. Biochim. Biophys. Acta.

[21] Peters A.H., O’Carroll D., Scherthan H., Mechtler K., Sauer S., Schöfer C., Weipoltshammer K., Pagani M., Lachner M., Kohlmaier A., Opravil S. (2001). Loss of the Suv39h histone methyltransferases impairs mammalian heterochromatin and genome stability. Cell.

[22] Peng J.C., Karpen G.H. (2007). H3K9 methylation and RNA interference regulate nucleolar organization and repeated DNA stability. Nat. Cell Biol..

[23] Sultana T., Zamborlini A., Cristofari G., Lesage P. (2017). Integration site selection by retroviruses and transposable elements in eukaryotes. Nat. Rev. Genet..

[24] Bulut-Karslioglu A., Inti A., Ramirez F., Barenboim M., Onishi-Seebacher M., Arand J., Galán C., Winter G.E., Engist B., Gerle B. (2014). Suv39h-dependent H3K9me3 marks intact retrotransposons and silences LINE elements in mouse embryonic stem cells. Mol. Cell.

[25] Pezic D., Manakov S.A., Sachidanandam R., Aravin A.A. (2014). piRNA pathway targets active LINE1 elements to establish the repressive H3K9me3 mark in germ cells. Genes Dev..

[26] Alexiadis V., Ballestas M.E., Sanchez C., Winokur S., Vedanarayanan V., Warren M., Ehrlich M. (2007). RNAPol-ChIP analysis of transcription from FSHD-linked tandem repeats and satellite DNA. Biochim. Biophys. Acta.

[27] Enukashvily N.I., Donev R., Waisertreiger I.R., Podgornaya O.I. (2007). Human chromosome 1 satellite 3 DNA is decondensed, demethylated and transcribed in senescent cells and in A431 epithelial carcinoma cells. Cytogenet. Genome Res..

[28] Frescas D., Guardavaccaro D., Kuchay S.M., Kato H., Poleshko A., Basrur V., Elenitoba-Johnson K.S., Katz R.A., Pagano M. (2008). KDM2A represses transcription of centromeric satellite repeats and maintains the heterochromatic state. Cell Cycle.

[29] Zhu Q., Pao G.M., Huynh A.M., Suh H., Tonnu N., Nederlof P.M., Gage F.H., Verma I.M. (2011). BRCA1 tumour suppression occurs via heterochromatin-mediated silencing. Nature.

[30] Collins N., Poot R.A., Kukimoto I., García-Jiménez C., Dellaire G., Varga-Weisz P.D. (2002). An ACF1-ISWI chromatin-remodeling complex is required for DNA replication through heterochromatin. Nat. Genet..

[31] Rowbotham S.P., Barki L., Neves-Costa A., Santos F., Dean W., Hawkes N., Choudhary P., Will W.R., Webster J., Oxley D. (2011). Maintenance of silent chromatin through replication requires SWI/SNF-like chromatin remodeler SMARCAD1. Mol. Cell.

[32] Neves-Costa A., Will W.R., Vetter A.T., Miller J.R., Varga-Weisz P. (2009). The SNF2-family member Fun30 promotes gene silencing in heterochromatic loci. PLoS ONE.

[33] Pegoraro G., Kubben N., Wickert U., Göhler H., Hoffmann K., Misteli T. (2009). Ageing-related chromatin defects through loss of the NURD complex. Nat. Cell Biol..

[34] Lindahl T., Barnes D.E. (2000). Repair of endogenous DNA damage. Cold Spring Harb. Symp. Quant. Biol..

[35] De Bont R., van Larebeke N. (2004). Endogenous DNA damage in humans: A review of quantitative data. Mutagenesis.

[36] Marnett L.J., Plastaras J.P. (2001). Endogenous DNA damage and mutation. Trends Genet..

[37] Tubbs A., Nussenzweig A. (2017). Endogenous DNA Damage as a Source of Genomic Instability in Cancer. Cell.

[38] Luijsterburg M.S., van Attikum H. (2011). Chromatin and the DNA damage response: The cancer connection. Mol. Oncol..

[39] Jiricny J. (2006). The multifaceted mismatch-repair system. Nat. Rev. Mol. Cell Biol..

[40] Scharer O.D. (2013). Nucleotide excision repair in eukaryotes. Cold Spring Harb. Perspect. Biol..

[41] Ceccaldi R., Sarangi P., D’Andrea A.D. (2016). The Fanconi anaemia pathway: New players and new functions. Nat. Rev. Mol. Cell Biol..

[42] Lieber M.R. (2010). The mechanism of double-strand DNA break repair by the nonhomologous DNA end-joining pathway. Annu. Rev. Biochem..

[43] Heyer W.D., Ehmsen K.T., Liu J. (2010). Regulation of homologous recombination in eukaryotes. Annu. Rev. Genet..

[44] Schuster-Bockler B., Lehner B. (2012). Chromatin organization is a major influence on regional mutation rates in human cancer cells. Nature.

[45] Zheng C.L., Wang N.J., Chung J., Moslehi H., Sanborn J.Z., Hur J.S., Collisson E.A., Vemula S.S., Naujokas A., Chiotti K.E. (2014). Transcription restores DNA repair to heterochromatin, determining regional mutation rates in cancer genomes. Cell Rep..

[46] Misteli T. (2007). Beyond the sequence: Cellular organization of genome function. Cell.

[47] Supek F., Lehner B. (2015). Differential DNA mismatch repair underlies mutation rate variation across the human genome. Nature.

[48] Adam S., Dabin J., Polo S.E. (2015). Chromatin plasticity in response to DNA damage: The shape of things to come. DNA Repair.

[49] Smerdon M.J., Lieberman M.W. (1978). Nucleosome rearrangement in human chromatin during UV-induced DNA- reapir synthesis. Proc. Natl. Acad. Sci. USA.

[50] Timinszky G., Till S., Hassa P.O., Hothorn M., Kustatscher G., Nijmeijer B., Colombelli J., Altmeyer M., Stelzer E.H., Scheffzek K. (2009). A macrodomain-containing histone rearranges chromatin upon sensing PARP1 activation. Nat. Struct. Mol. Biol..

[51] Khurana S., ruhlak M.J., Kim J., Tran A.D., Liu J., Nyswaner K., Shi L., Jailwala P., Sung M.H., Hakim O. (2014). A macrohistone variant links dynamic chromatin compaction to BRCA1-dependent genome maintenance. Cell Rep..

[52] Di Micco R., Sulli G., Dobreva M., Liontos M., Botrugno O.A., Gargiulo G., dal Zuffo R., Matti V., d’Ario G., Montani E. (2011). Interplay between oncogene-induced DNA damage response and heterochromatin in senescence and cancer. Nat. Cell Biol..

[53] Murga M., Jaco I., Fan Y., Soria R., Martinez-Pastor B., Cuadrado M., Yang S.M., Blasco M.A., Skoultchi A.I., Fernandez-Capetillo O. (2007). Global chromatin compaction limits the strength of the DNA damage response. J. Cell Biol..

[54] Iacovoni J.S., Caron P., Lassadi I., Nicolas E., Massip L., Trouche D., Legube G. (2010). High-resolution profiling of gammaH2AX around DNA double strand breaks in the mammalian genome. EMBO J..

[55] Sabarinathan R., Mularoni L., Deu-Pons J., Gonzalez-Perez A., Lopez-Bigas N. (2016). Nucleotide excision repair is impaired by binding of transcription factors to DNA. Nature.

[56] Perera D., Poulos R.C., Shah A., Beck D., Pimanda J.E., Wong J.W. (2016). Differential DNA repair underlies mutation hotspots at active promoters in cancer genomes. Nature.

[57] Polak P., Lawrence M.S., Haugen E., Stoletzki N., Stojanov P., Thurman R.E., Garraway L.A., Mirkin S., Getz G. (2014). Reduced local mutation density in regulatory DNA of cancer genomes is linked to DNA repair. Nat. Biotechnol..

[58] Pleasance E.D., Cheetham R.K., Stephens P.J., McBride D.J., Humphray S.J., Greenman C.D., Varela I., Lin M.L., Ordóñez G.R., Bignell G.R. (2010). A comprehensive catalogue of somatic mutations from a human cancer genome. Nature.

[59] Stamatoyannopoulos J.A., Adzhubei I., Thurman R.E., Kryukov G.V., Mirkin S.M., Sunyaev S.R. (2009). Human mutation rate associated with DNA replication timing. Nat. Genet..

[60] Knezetic J.A., Luse D.S. (1986). The presence of nucleosomes on a DNA template prevents initiation by RNA polymerase II in vitro. Cell.

[61] Han M., Grunstein M. (1988). Nucleosome loss activates yeast downstream promoters in vivo. Cell.

[62] Kayne P.S., Him U.J., Han M., Mullen J.R., Yoshizaki F., Grunstein M. (1988). Extremely conserved histone H4 N terminus is dispensable for growth but essential for repressing the silent mating loci in yeast. Cell.

[63] Kouzarides T. (2007). Chromatin modifications and their function. Cell.

[64] Fragkos M., Ganier O., Coulombe P., Méchali M. (2015). DNA replication origin activation in space and time. Nat. Rev. Mol. Cell Biol..

[65] MacAlpine D.M., Almouzni G. (2013). Chromatin and DNA replication. Cold Spring Harb. Perspect. Biol..

[66] Kurat C.F., Yeeles J.T., Patel H., Early A., Diffley J.F. (2017). Chromatin Controls DNA Replication Origin Selection, Lagging-Strand Synthesis, and Replication Fork Rates. Mol. Cell.

[67] Devbhandari S., Jiang J., Kumar C., Whitehouse I., Remus D. (2017). Chromatin Constrains the Initiation and Elongation of DNA Replication. Mol. Cell.

[68] Rivera-Mulia J.C., Gilbert D.M. (2016). Replicating Large Genomes: Divide and Conquer. Mol. Cell.

[69] Pope B.D., Ryba T., Dileep V., Yue F., Wu W., Denas O., Vera D.L., Wang Y., Hansen R.S., Canfield T.K. (2014). Topologically associating domains are stable units of replication-timing regulation. Nature.

[70] Dileep V., Ay F., Sima J., Vera D.L., Noble W.S., Gilbert D.M. (2015). Topologically associating domains and their long-range contacts are established during early G1 coincident with the establishment of the replication-timing program. Genome Res..

[71] Foti R., Gnan S., Cornacchia D., Dileep V., Bulut-Karslioglu A., Diehl S., Buness A., Klein F.A., Huber W., Johnstone E. (2016). Nuclear Architecture Organized by Rif1 Underpins the Replication-Timing Program. Mol. Cell.

[72] Beck H., Nähse-Kumpf V., Larsen M.S.Y., O’Hanlon K.A., Patzke S., Holmberg C., Mejlvang J., Groth A., Nielsen O., Syljuåsen R.G. (2012). Cyclin-dependent kinase suppression by WEE1 kinase protects the genome through control of replication initiation and nucleotide consumption. Mol. Cell Biol..

[73] Venkatesh S., Workman J.L. (2015). Histone exchange, chromatin structure and the regulation of transcription. Nat. Rev. Mol. Cell Biol..

[74] Schwabish M.A., Struhl K. (2006). Asf1 mediates histone eviction and deposition during elongation by RNA polymerase II. Mol. Cell.

[75] Kaplan C.D., Laprade L., Winston F. (2003). Transcription elongation factors repress transcription initiation from cryptic sites. Science.

[76] Aguilera A., Garcia-Muse T. (2012). R loops: From transcription byproducts to threats to genome stability. Mol. Cell.

[77] Gaillard H., Aguilera A. (2016). Transcription as a Threat to Genome Integrity. Annu. Rev. Biochem..

[78] Skourti-Stathaki K., Proudfoot N.J. (2014). A double-edged sword: R loops as threats to genome integrity and powerful regulators of gene expression. Genes Dev..

[79] Li X., Manley J.L. (2006). Cotranscriptional processes and their influence on genome stability. Genes Dev..

[80] Sankar T.S., Wastuwidyaningtyas B.D., Dong Y., Lewis S.A., Wang J.D. (2016). The nature of mutations induced by replication-transcription collisions. Nature.

[81] Aguilera A. (2002). The connection between transcription and genomic instability. EMBO J..

[82] Helmrich A., Ballarino M., Nudler E., Tora L. (2013). Transcription-replication encounters, consequences and genomic instability. Nat. Struct. Mol. Biol..

[83] Morganella S., Alexandrov L.B., Glodzik D., Zou X., Davies H., Staaf J., Sieuwerts A.M., Brinkman A.B., Martin S., Ramakrishna M. (2016). The topography of mutational processes in breast cancer genomes. Nat. Commun..

[84] Sima J., Gilbert D.M. (2014). Complex correlations: Replication timing and mutational landscapes during cancer and genome evolution. Curr. Opin. Genet. Dev..

[85] Noon A.T., Shibata A., Rief N., Löbrich M., Stewart G.S., Jeggo P.A., Goodarzi A.A. (2010). 53BP1-dependent robust localized KAP-1 phosphorylation is essential for heterochromatic DNA double-strand break repair. Nat. Cell Biol..

[86] Rubbi C.P., Milner J. (2003). p53 is a chromatin accessibility factor for nucleotide excision repair of DNA damage. EMBO J..

[87] Kruhlak M.J., Celeste A., Dellaire G., Fernandez-Capetillo O., Müller W.G., McNally J.G., Bazett-Jones D.P., Nussenzweig A. (2006). Changes in chromatin structure and mobility in living cells at sites of DNA double-strand breaks. J. Cell Biol..

[88] Price B.D., D’Andrea A.D. (2013). Chromatin remodeling at DNA double-strand breaks. Cell.

[89] Hauer M.H., Seeber A., Singh V., Thierry R., Sack R., Amitai A., Kryzhanovska M., Eglinger J., Holcman D., Owen-Hughes T. (2017). Histone degradation in response to DNA damage enhances chromatin dynamics and recombination rates. Nat. Struct. Mol. Biol..

[90] Berkovich E., Monnat R.J., Kastan M.B. (2007). Roles of ATM and NBS1 in chromatin structure modulation and DNA double-strand break repair. Nat. Cell Biol..

[91] Goldstein M., Derheimer F.A., Tait-Mulder J., Kastan M.B. (2013). Nucleolin mediates nucleosome disruption critical for DNA double-strand break repair. Proc. Natl. Acad. Sci. USA.

[92] Falk M., Lukasova E., Gabrielova B., Ondrej V., Kozubek S. (2007). Chromatin dynamics during DSB repair. Biochim. Biophys. Acta.

[93] Murr R., Loizou J.I., Yang Y.G., Cuenin C., Li H., Wang Z.Q., Herceg Z. (2006). Histone acetylation by Trrap–Tip60 modulates loading of repair proteins and repair of DNA double-strand breaks. Nat. Cell Biol..

[94] Xu Y., Jiang X., Ayrapetov M.K., Moskwa P., Yang S., Price B.D. (2010). The p400 ATPase regulates nucleosome stability and chromatin ubiquitination during DNA repair. J. Cell Biol..

[95] Xu Y., Ayrapetov M.K., Xu C., Gursoy-Yuzugullu O., Hu Y., Price B.D. (2012). Histone H2A.Z controls a critical chromatin remodeling step required for DNA double-strand break repair. Mol. Cell.

[96] Sobhian B., Shao G., Lilli D.R., Culhane A.C., Moreau L.A., Xia B., Greenberg R.A. (2007). RAP80 targets BRCA1 to specific ubiquitin structures at DNA damage sites. Science.

[97] Luijsterburg M.S., Typas D., Caron M.C., Wiegant W.W., van den Heuvel D., Boonen R.A., Couturier A.M., Mullenders L.H., Masson J.Y., van Attikum H. (2017). A PALB2-interacting domain in RNF168 couples homologous recombination to DNA break-induced chromatin ubiquitylation. eLife.

[98] Botuyan M.V., Lee J., Ward I.M., Kim J.E., Thompson J.R., Chen J., Mer G. (2006). Structural basis for the methylation state-specific recognition of histone H4-K20 by 53BP1 and Crb2 in DNA repair. Cell.

[99] Luijsterburg M.S., de Krijger I., Wiegant W.W., Shah R.G., Smeenk G., de Groot A.J., Pines A., Vertegaal A.C., Jacobs J.J., Shah G.M. (2016). PARP1 Links CHD2-Mediated Chromatin Expansion and H3.3 Deposition to DNA Repair by Non-homologous End-Joining. Mol. Cell.

[100] Wang H., Zhai L., Xu J., Joo H.Y., Jackson S., Erdjument-Bromage H., Tempst P., Xiong Y., Zhang Y. (2006). Histone H3 and H4 ubiquitylation by the CUL4-DDB-ROC1 ubiquitin ligase facilitates cellular response to DNA damage. Mol. Cell.

[101] Jorgensen S., Eskildsen M., Fugger K., Hansen L., Larsen M.S.Y., Kousholt A.N., Syljuåsen R.G., Trelle M.B., Jensen O.N. (2011). SET8 is degraded via PCNA-coupled CRL4(CDT2) ubiquitylation in S phase and after UV irradiation. J. Cell Biol..

[102] Jorgensen S., Schotta G., Sorensen C.S. (2013). Histone H4 lysine 20 methylation: Key player in epigenetic regulation of genomic integrity. Nucleic Acids Res..

[103] Schotta G., Lachner M., Sarma K., Ebert A., Sengupta R., Reuter G., Reinberg D., Jenuwein T. (2004). A silencing pathway to induce H3-K9 and H4-K20 trimethylation at constitutive heterochromatin. Genes Dev..

[104] Schotta G., Sengupta R., Kubicek S., Malin S., Kauer M., Callén E., Celeste A., Pagani M., Opravil S., Inti A. (2008). A chromatin-wide transition to H4K20 monomethylation impairs genome integrity and programmed DNA rearrangements in the mouse. Genes Dev..

[105] Ayoub N., Jeyasekharan A.D., Bernal J.A., Venkitaraman A.R. (2008). HP1-β mobilization promotes chromatin changes that initiate the DNA damage response. Nature.

[106] Warters R.L., Lyons B.W. (1992). Variation in radiation-induced formation of DNA double-strand breaks as a function of chromatin structure. Radiat. Res..

[107] Elia M.C., Bradley M.O. (1992). Influence of chromatin structure on the induction of DNA double strand breaks by ionizing radiation. Cancer Res..

[108] Yi X., de Vries H.I., Siudeja K., Rana A., Lemstra W., Brunsting J.F., Kok R.M., Smulders Y.M., Schaefer M., Dijk F. (2009). Stwl modifies chromatin compaction and is required to maintain DNA integrity in the presence of perturbed DNA replication. Mol. Biol. Cell.

[109] Cann K.L., Dellaire G. (2011). Heterochromatin and the DNA damage response: The need to relax. Biochem. Cell Biol..

[110] Falk M., Lukasova E., Kozubek S. (2008). Chromatin structure influences the sensitivity of DNA to gamma-radiation. Biochim. Biophys. Acta.

[111] Ayrapetov M.K., Gursoy-Yuzugullu O., Xu C., Xu Y., Price B.D. (2014). DNA double-strand breaks promote methylation of histone H3 on lysine 9 and transient formation of repressive chromatin. Proc. Natl. Acad. Sci. USA.

[112] Kaidi A., Jackson S.P. (2013). KAT5 tyrosine phosphorylation couples chromatin sensing to ATM signalling. Nature.

[113] Goodarzi A.A., Kurka T., Jeggo P.A. (2011). KAP-1 phosphorylation regulates CHD3 nucleosome remodeling during the DNA double-strand break response. Nat. Struct. Mol. Biol..

[114] Janssen A., Breuer G.A., Brinkman E.K., van der Meulen A.I., Borden S.V., van Steensel B., Bindra R.S., LaRocque J.R., Karpen G.H. (2016). A single double-strand break system reveals repair dynamics and mechanisms in heterochromatin and euchromatin. Genes Dev..

[115] Chapman J.R., Taylor M.R., Boulton S.J. (2012). Playing the end game: DNA double-strand break repair pathway choice. Mol. Cell.

[116] Sonoda E., Hochegger H., Saberi A., Taniguchi Y., Takeda S. (2006). Differential usage of non-homologous end-joining and homologous recombination in double strand break repair. DNA Repair.

[117] Pfister S.X., Ahrabi S., Zalmas L.P., Sarkar S., Aymard F., Bachrati C.Z., Helleday T., Legube G., La Thangue N.B., Porter A.C. (2014). SETD2-dependent histone H3K36 trimethylation is required for homologous recombination repair and genome stability. Cell Rep..

[118] Gong F., Miller K.M. (2013). Mammalian DNA repair: HATs and HDACs make their mark through histone acetylation. Mutat. Res..

[119] Courilleau C., Chailleux C., Jauneau A., Grimal F., Briois S., Boutet-Robinet E., Boudsocq F., Trouche D., Canitrot Y. (2012). The chromatin remodeler p400 ATPase facilitates Rad51-mediated repair of DNA double-strand breaks. J. Cell Biol..

[120] Tang J., Cho N.W., Cui G., Manion E.M., Shanbhag N.M., Botuyan M.V., Mer G., Greenberg R.A. (2013). Acetylation limits 53BP1 association with damaged chromatin to promote homologous recombination. Nat. Struct. Mol. Biol..

[121] Becker A., Durante M., Taucher-Scholz G., Jakob B. (2014). ATM alters the otherwise robust chromatin mobility at sites of DNA double-strand breaks (DSBs) in human cells. PLoS ONE.

[122] Kalousi A., Hoffbeck A.S., Selemenakis P.N., Pinder J., Savage K.I., Khanna K.K., Brino L., Dellaire G., Gorgoulis V.G., Soutoglou E. (2015). The nuclear oncogene SET controls DNA repair by KAP1 and HP1 retention to chromatin. Cell Rep..

[123] Lee Y.H., Kuo C.Y., Stark J.M., Shih H.M., Ann D.K. (2013). HP1 promotes tumor suppressor BRCA1 functions during the DNA damage response. Nucleic Acids Res..

[124] Soria G., Almouzni G. (2013). Differential contribution of HP1 proteins to DNA end resection and homology-directed repair. Cell Cycle.

[125] Geuting V., Reul C., Lobrich M. (2013). ATM release at resected double-strand breaks provides heterochromatin reconstitution to facilitate homologous recombination. PLoS Genet..

[126] Brandsma I., Gent D.C. (2012). Pathway choice in DNA double strand break repair: Observations of a balancing act. Genome Integr..

[127] Bakkenist C.J., Kastan M.B. (2003). DNA damage activates ATM through intermolecular autophosphorylation and dimer dissociation. Nature.

[128] Fan Y., Nikitina T., Zhao J., Fleury T.J., Bhattacharyya R., Bouhassira E.E., Stein A., Woodcock C.L., Skoultchi A.I. (2005). Histone H1 depletion in mammals alters global chromatin structure but causes specific changes in gene regulation. Cell.

[129] Bao S., Wu Q., McLendon R.E., Hao Y., Shi Q., Hjelmeland A.B., Dewhirst M.W., Bigner D.D., Rich J.N. (2006). Glioma stem cells promote radioresistance by preferential activation of the DNA damage response. Nature.

[130] Meshorer E., Yellajoshula D., George E., Scambler P.J., Brown D.T., Misteli T. (2006). Hyperdynamic plasticity of chromatin proteins in pluripotent embryonic stem cells. Dev. Cell.

[131] Burgess R.C., Burman B., Kruhlak M.J., Misteli T. (2014). Activation of DNA damage response signaling by condensed chromatin. Cell Rep..

[132] Soutoglou E., Misteli T. (2008). Activation of the cellular DNA damage response in the absence of DNA lesions. Science.

[133] Olcina M.M., Foskolou I.P., Anbalagan S., Senra J.M., Pires I.M., Jiang Y., Ryan A.J., Hammond E.M. (2013). Replication stress and chromatin context link ATM activation to a role in DNA replication. Mol. Cell.

